# Sequential primary cutaneous follicle center lymphoma and marginal zone B-cell lymphoma arising in the same patient

**DOI:** 10.1016/j.jdcr.2023.07.028

**Published:** 2023-08-03

**Authors:** Gabriella Alvarez, Larissa Rodriguez-Homs, Rami N. Al-Rohil, Meenal Kheterpal, Amber Fresco

**Affiliations:** a Duke University School of Medicine, Durham, North Carolina; b Department of Dermatology, Duke University, Durham, North Carolina; c Department of Pathology, Duke University Hospital, Durham, North Carolina

**Keywords:** cutaneous neoplasm, lymphoma, PCFCL, PCMZL

## Introduction

Primary cutaneous follicle center lymphoma (PCFCL) is a low-grade B-cell lymphoproliferative neoplasm limited to the skin. PCFCL accounts for 50% of all cutaneous B-cell lymphomas and generally has an excellent prognosis.^1^ Primary cutaneous marginal zone B-cell lymphoproliferative disorder (PCMZLPD) recurs commonly but shows extremely indolent behavior and does not require aggressive therapies, and the disease-specific survivals approach 100%. PCMZLPD accounts for about 2% to 16% of all cutaneous atypical hematolymphoid infiltrates.^2^ In the following case, a young, adult woman presented to dermatology clinic for evaluation of a presumed keloid. She was instead found to have PCFCL and later developed new lesions consistent with PCMZLPD. The co-occurrence of these 2 cutaneous low-grade B-cell neoplasms in a single patient is unusual with limited cases in the literature. Herein, we discuss the workup and diagnosis of primary cutaneous B-cell lymphomas based on a rare case.

## Case report

A 24-year-old woman with past medical history of polycystic ovarian syndrome presented to dermatology clinic for evaluation of a lesion referred by primary care. She reported that the lesion on her right shoulder was present for 2 years. It had grown and was pruritic. She denied associated trauma, pain, bleeding, or use of topical creams. She also denied history of prior keloids. The review of systems was negative for unintentional weight loss, fever, chills, or night sweats. Her only medication at this time was an oral contraceptive.

**Fig 1:**
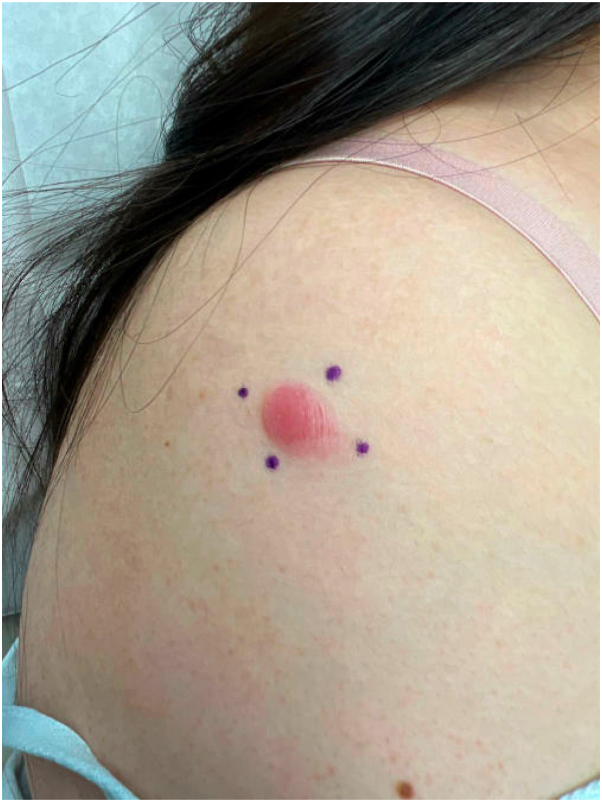
Patient images show a shiny, bright pink nodule on the right lateral shoulder at the time of biopsy.

**Fig 2:**
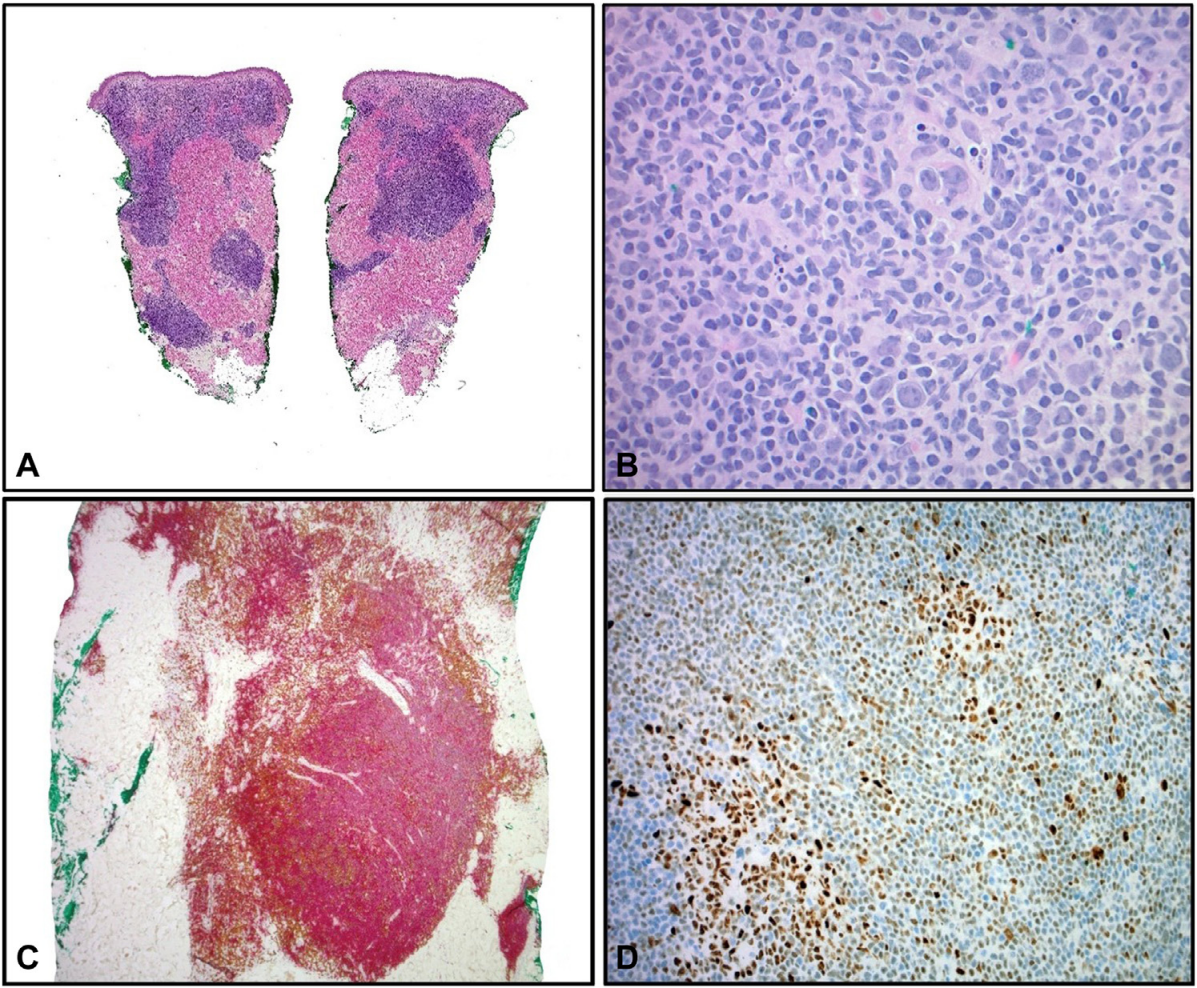
Pathology: Hematoxylin-eosin and immunohistochemical stains. Skin punch biopsy from the right lateral shoulder shows a diffuse dermal lymphoid infiltrate with no definitive germinal center formation (**A,** original magnification: ×2). The infiltrate is composed of a mixture of mostly small sized cleaved cells showing irregular shaped nuclei admixed with scattered larger cells showing multiple nucleoli (*centroblasts*) (**B,** original magnification: ×40). Immunohistochemically, the lymphoid infiltrate is mostly composed of sheets of CD20^+^ B cells (*red chromogen*) with admixed CD3^+^ T cells (*brown chromogen*) (**C,** CD3/CD20 cocktail; original magnification: ×4). The B cells show aberrant expression of BCL-6 (**D,** BCL-6; original magnification: ×20).

Her physical examination revealed a 2-cm soft, shiny, pink nodule on the right lateral shoulder (Fig 1). Given the lack of induration, which is expected from a keloid, a punch biopsy was performed to clarify the diagnosis. Pathology revealed a diffuse dermal lymphoid infiltrate showing no epidermal involvement and lacking germinal center formation (Fig 2, *A*). The cells were composed of small, irregularly shaped lymphocytes with scattered larger forms displaying multiple nucleoli (Fig 2, *B*). The atypical cells were diffusely immunoreactive for CD20 and BCL-2, and had variable expression of BCL-6, with BCL-6 being expressed in the majority of the B cells that coexpress BCL-2 (Fig 2, *C*, *D*). Kappa and lambda stains by in situ hybridization showed polytypic plasma cells, whereas MUM-1 and CD10 were negative. The findings were consistent with low-grade B-cell lymphoma, follicle center immunophenotype. Next generation sequencing for B-cell clonality was positive for Ig Kappa. The differential diagnosis included PCFCL vs secondary cutaneous involvement by systemic follicle center lymphoma.

Workup included a positron emission tomography imaging showing no evidence of avid lymphoma. The patient had mild leukocytosis with no lymphocytosis; thus, there was no indication for bone marrow biopsy. Colonoscopy and peripheral blood flow cytometry were both unremarkable. Given the low risk of systemic involvement, she was diagnosed with PCFCL. Her lesion was treated with radiation therapy with a dose of 30 Gy with resolution.

**Fig 3:**
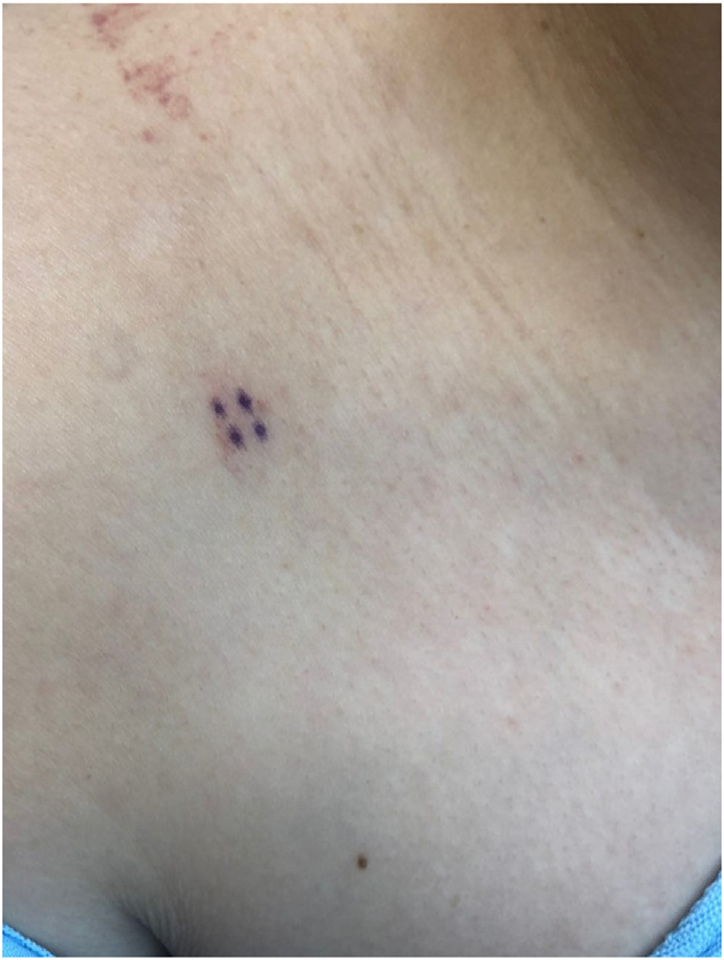
Patient images on second presentation. The image of a new lesion on the right medial shoulder (primary cutaneous marginal zone B-cell lymphoma) that appeared months after her primary cutaneous follicle center lymphoma diagnosis.

**Fig 4:**
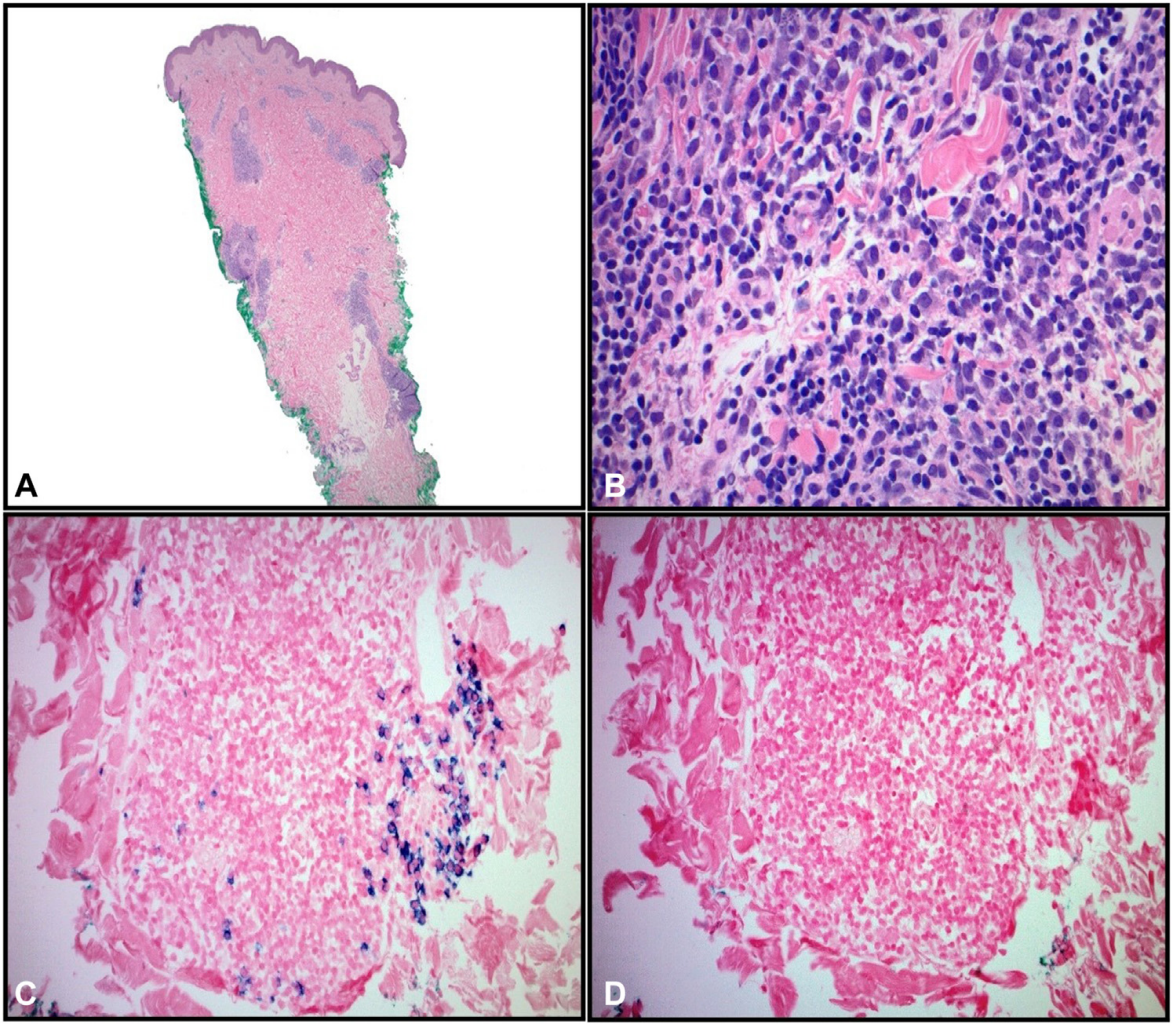
Pathology: Hematoxylin-eosin, lambda, and kappa stains. On low power, the right medial shoulder biopsy shows a dermal-based lymphoid infiltrate that tracked along the adnexal structures (**A,** hematoxylin-eosin stain; original magnification: ×2). On closer inspection, the infiltrate is composed of monotonous centrocyte-like cells with plasmacytoid and plasma cells (**B,** hematoxylin-eosin stain; original magnification: ×40). By colorimetric in situ hybridization, the plasma cells are lambda-restricted (**C,** lambda stain; original magnification: ×20) but completely negative for kappa (**D,** kappa stain; original magnification: ×20) (*kappa is negative*).

Five months after the treatment, she presented again with a new pink macule on the right medial shoulder (Fig 3). Repeat biopsy revealed atypical dermal lymphoid infiltrates with scattered plasma and plasmacytoid cells consistent with PCMZLPD (Fig 4, *A*, *D*). B cells expressed BCL-2. The plasma cells were lambda-restricted. Next-generation sequencing for B-cell clonality was positive Ig Kappa. The sequence of this clonal product was different from that detected in the patient’s right lateral shoulder biopsy. Given the presence of a new primary malignancy, she was restaged with positron emission tomography imaging (negative), and full body skin examination on the following month was negative. She was treated with 24 Gy/12 fractions to the new lesion.

Three months after the PCMZLPD diagnosis, she presented with yet another lesion on the posterior right shoulder, described as a 1.5-cm pink, ill-defined patch. Biopsy revealed findings similar to the lesion on her right medial shoulder. Additional workup included Lyme screening (negative). The treatment of this lesion included a combination of intralesional triamcinolone and topical clobetasol ointment.

## Discussion

PCFCL is the most common subtype of primary cutaneous B-cell lymphomas, representing 10% to 20% of all cutaneous lymphomas. Clinical presentation of PCFCL is solitary or grouped pink-to-violaceous papules, plaques, or nodules typically found on the head and neck regions. Differential diagnoses include acne, epidermal inclusion cysts, arthropod bites, cutaneous lymphoid hyperplasia, and other cutaneous neoplasms.^3^ PCFCL shows abnormal lymphocyte morphology with monotonous cleaved cells (centrocytes) and larger cells with vesicular chromatin and multiple nucleoli (centroblasts).^4^ Compared to cutaneous lymphoid hyperplasia, germinal centers are either absent (diffuse type) or show abnormal morphology (follicular type), represented by a monomorphic appearance with no zonation, reduced/absent mantle zone, and absence of tingible body macrophages.^5^ Main differentiating features are the presence of aberrant BCL-2 and BCL-6 expression. Molecular testing shows monoclonal rearrangement of immunoglobulin genes in most cases.

PCMZLPDs are indolent, reaching almost 100% survival rate without aggressive therapy.^2^^,^^6^ The nomenclature has been changed because primary cutaneous form is much different from other sites of mucosa associated lymphoid tumor lymphomas.^6^ Clinical presentation includes deep red-to-violaceous infiltrated plaques, nodules, or tumors with surrounding diffuse or annular erythema on the trunk or extremities.^5^ Differential diagnoses include benign cutaneous lymphoproliferation and primary or secondary cutaneous B or T-cell lymphomas.^7^ Neoplastic B cells are typically BCL-2^+^, BCL6^−^, and CD10^−^, which helps differentiate PCMZLPD from PCFCL (BCL6^+^) and cutaneous lymphoid hyperplasia.^2^ The plasma and lymphoplasmacytoid cells are monoclonal and restricted for either Kappa or Lambda. There have been associations with chromosomal translocations t(14;18)(q32;q21), immunoglobulin heavy-chain locus, mucosa associated lymphoid tissue 1 gene, and trisomy 18.^2^^,^^7^ There are also associations with *Borrelia burgdorferi* in endemic areas (screened negative).^2^^,^^7^^,^^8^

It is rare to have sequential primary lymphomas in a single patient. One study found that the most common sequential primary lymphomas were diffuse large B-cell lymphoma and adult T-cell leukemia in 3 of 49 patients. However, that same study did identify one case of dual diagnosis of PCFCL and PCMZLPD.^9^ Our patient was originally diagnosed with PCFCL, and within months, she developed 2 subsequent lesions consistent with PCMZLPD. This case adds to the little existing knowledge, which may encourage the providers to perform biopsies on new lesions even after a primary lesion has been identified.

## Conflicts of interest

None disclosed.
